# Arbuscular Mycorrhizal Fungi Promote Soil Respiration Primarily Through Mediating Microbial and Root Biomass in Rocky Desertification Habitat

**DOI:** 10.3390/jof11090616

**Published:** 2025-08-24

**Authors:** Shuang Zhao, Shaojun Wang, Yali Song, Lingling Xie, Bo Xiao, Xiaofei Guo

**Affiliations:** 1 Yunnan Key Laboratory of Plateau Wetland Conservation, Restoration and Ecological Services, National Plateau Wetlands Research Center, Southwest Forestry University, 300 Bailongsi, Kunming 650224, China; zhaoshuang686883@163.com (S.Z.); xielingling1999@163.com (L.X.); xiaobo5751@163.com (B.X.); 2 College of forestry, Guangxi University, Nanning 530007, China; 3 College of Soil and Water Conservation, Southwest Forestry University, 300 Bailongsi, Kunming 650224, China; guoxiaofeins@163.com

**Keywords:** arbuscular mycorrhizal fungi, symbiosis, soil respiration, microbial and root biomass, rocky desertification

## Abstract

Arbuscular mycorrhizal (AM) fungi can have complicated interactions with plants and soils, which play a critical role in mediating the soil carbon cycle. However, the mechanism by which AM fungi regulate soil respiration is not well documented. This study conducted a completely randomized block-design mesocosm experiment using the inoculation of AM fungi (RI: *Rhizophagus intraradices*; FM: *Funneliformis mosseae*) with *Fraxinus malacophylla* to identify the pathways of AM fungi controlling soil respiration in a rocky desertification habitat. We observed that the average soil respiration rates (3.78 μmol·m^−2^·s^−1^) were significantly higher in two AM fungi inoculation treatments than in the control (2.87 μmol·m^−2^·s^−1^). Soil respiration rates were 1.59-fold higher in RI fungi inoculation and 1.05-fold higher in FM inoculation than in the control. Explanation rates of microbial biomass carbon, biomass nitrogen, and root biomass in RI (57.46–76.49%) and FM (44.81–62.62%) inoculation for soil respiration variation were higher than those in the control (24.51–34.32%). The direct positive pathway of soil respiration was mainly regulated by microbial biomass (59.5%) and root biomass (34.90%), while the indirect positive contributions of soil physicochemical properties (30.00%), colonization level (3.50%), soil microclimate (19.30%), and enzyme activity (3.38%) to respiration dynamics ranked second. Thus, we conclude that soil respiration dynamics can be mainly controlled by AM fungi-mediated changes in microbial and root biomass in rocky desertification areas.

## 1. Introduction

In recent decades, the substantial increase in the level of atmospheric greenhouse gases such as CO_2_ has greatly accelerated the process of global warming [1]. Soil plays an important role in the composition of terrestrial carbon reservoirs, storing about 3000 Pg of carbon within a depth of 1 m [2]. Soil respiration is a main pathway of ecosystem carbon returning into the atmosphere via CO_2_ emissions [3,4], which may play a critical role in mediating the carbon sequestration in soils. Small changes in soil respiration rates can exert pivotal effects on CO_2_ concentrations in the atmosphere [5,6]. Therefore, it is important to identify the mechanism by which the variation in soil respiration rates can be controlled.

Most of the studies focus on the abiotic factors that affect soil respiration [7]; however, little is known about how the biotic factors regulate the soil respiration process. Arbuscular mycorrhizal (AM) fungi can form a mutualistic symbiosis with more than 80% of the world’s plants [8], which affects root growth as well as microbial and faunal activities through modifying soil physical and chemical environments [9]. This would stimulate microbial, faunal, and root respiration [10]. Furthermore, AM fungi may have a greater effect on soil respiration through accelerating plant growth and soil nutrient availability in the nutrient-limited areas [11]. In particular, the impact of AM fungi on the soil respiration rates varies with their species due to their differential modifications of plant, enzymatic, and soil properties. Therefore, it is critical to identify the pathways by which AM fungi can mediate soil respiration dynamics.

The world’s largest karst areas are mainly distributed in southern China [12]. The karst desertification had a low soil carbon cycling rate due to a high ratio of bare stone to shallow soil, low plant cover, poor soil nutrients, and a lack of microbes and fauna [13]. The AM fungi are thus widely used to restore plants and soils in these areas [14,15]. However, few studies have focused on the mechanisms by which AM fungi affect the soil carbon cycling process in the stony desertification area. Therefore, a completely randomized block-design mesocosm experiment was conducted using the inoculation of AM fungi (RI: *Rhizophagus intraradices*; FM: *Funneliformis mosseae*) with *Fraxinus malacophylla* in a rocky desertification habitat in Mile city, Yunnan province, China. The objective of the research was to (i) determine temporal patterns of soil respiration characteristics under the inoculation of two AM fungi species; (ii) explore the linkage of soil respiration with plant growth, enzyme activity, and soil physicochemical properties; and (iii) identify the main path driving soil respiration under AM fungi inoculation in degraded karst areas.

## 2. Materials and Methods

### 2.1. Soil, AM Fungi, and Plant Preparations

The desertification soil was sampled from the top 0–20 cm layer of a stony karst land in the Mile Desertification Control Experimental Station [16]. The soil (0.95 mg kg^−1^ total N; 3.78 g kg^−1^ organic matter; 0.99 mg g^−1^ total P; 3.82 mg g ^−1^ available P (Olsen-P); 2.56 available K; 7.62 for the pH value (H_2_O, soil 2.5:1, g/mL)) was a calcareous agrotype developed from limestone. The soil was air-dried, sieved to pass 2 mm, and then fully mixed. A native AM species that has been widely proven to be distributed in the study area was selected as the inoculated strain. We referred to Wang et al. [17] to determine the inoculum dosage. Their inoculum (i.e., containing spores, external hyphae, and inoculated root fragments) of *R. intraradices* (BGC HEB07D) and *F. mosseae* (BGC NM01A) was acquired from the Bank of Glomales in China [18]. The spore concentrations were an inoculum of 144 spores g^−1^ evaluated by the wet sieving method [19].

The *F. Malacophylla* is a common plant species mainly distributed in the rocky desertification areas of Yunnan province. The seedlings used in the experiment were grown in the above soils from November 2019 to January 2020 in a greenhouse of the Mile Desertification Control Experimental Station (103°17′ E, 24°17′ N), Yunnan, southwestern China. Each (65 fine roots; 7 cm length per fine root) of the seedlings was free of disease and insects. Before inoculating, the seedlings were confirmed to have no AM spores or hyphae through the microscopy method, after being rinsed 3–4 times with aseptic water, surface-sterilized with 75% ethanol for 30 s, rinsed thoroughly with deionized water, and placed on an autoclaved filter paper soaked with sterile distilled water.

### 2.2. Experimental Design and Inoculation

The AM fungi were inoculated in a mesocosm (plastic pots; 50 cm diameter and 30 cm height) in the greenhouse of the Mile Desertification Control Experimental Station, Yunnan, southwestern China, from 3 February to 26 December 2020. Our study was conducted under natural precipitation conditions. During the experiment, the mean yearly air temperature and rainfall were 15 °C and 900–1000 mm, respectively. Mesocosms were filled with the above soil up to about 5 cm from the top. One seedling per mesocosm was planted in May, one month before the experiment started. The seedlings of *F. malacophylla* were inoculated with two AM fungi species (RI: *R. intraradices*; FM: *F. mosseae*). This experiment was carried out in a completely randomized block design and thus consisted of three treatments: (i) CK: without AM; (ii) RI: *R. intraradices* inoculation; and (iii) FM: *F. mosseae* inoculation. The 40 g AM inoculum (5760 spores) per kg soil was placed near the roots under the soil surface (10 cm soil depth) in AM treatments. Each treatment was replicated 24 times, resulting in a total of 72 mesocosms. In CK, 10 mL of a soil filtrate (0.45 μm pore size) and sterilized AM fungi were added to the pots to ensure that it was the same as the other two treatments.

### 2.3. Soil Respiration Measurement

The soil respiration rates were determined using a respiration chamber (Li 6400-09, Li Inc., Lincoln, NE, USA) that was connected to a portable infrared gas analyzer (Li Inc.) [20]. They were measured in a polyvinyl chloride (PVC) collar (5 cm diameter and 5 cm height) that was installed 2–3 cm into the soil near the roots of seedlings in mesocosms three days in advance to avoid the soil disturbance caused by the embedding of the PVC [21]. In March, June, September, and December 2020, the respiration rates were randomly measured twice (approx. day 15) each month for each of three treatments (RI, FM, and CK). Each measurement of soil respiration was taken between 9:00 and 11:00 h [2]. The temperature and water in 0–20 cm soil layers were measured simultaneously near respiration measurement locations by a convenient moisture–temperature meter (SINTN8, Shandong Lande Intelligent Technology Co., Ltd., Jinan, China).

### 2.4. Sampling and Determination

The height (H) and base diameter (BDH) of host seedlings in the three treatments were measured once every month with a vernier caliper and steel tape, respectively. In March, June, September, and December 2020, roots and rhizosphere soils were collected from six mesocosms of each experimental treatment. Roots were gently removed from the soil by handpicking and sieving (2 mm). AM colonization (RC) was measured by staining with Trypan blue [22]. For the measurement, fresh root samples (2 g) were cut into 1 cm root pieces, cleared in 10% KOH, acidified with 2% HCl, and stained with 0.05% Trypan blue in a 90 °C water bath for 30 min. After decolorization, 30 root segments were randomly selected and prepared as slides, and 10 roots were mounted onto each glass slide to observe for infections at 200× magnification under the microscope. The hyphal length density (HLD) in soils was measured by vacuum pump microporous filter extraction. In brief, 5 g rhizosphere soil samples were dispersed in deionized water (50 mL) and shaken for 30 s (in an end-over-end shaker). After 30 min, the suspension was decanted through 20 μm and 400 μm double-deck screens. Retained hyphae, roots, and organic matter in the 400 μm mesh sieve were transferred with 200 mL of deionized water into a 500 mL flask and shaken, and stained in 0.05% Trypan blue; finally, using the modified line intersect method at 200× magnification, hyphal length was estimated. The hyphal length data were converted to hyphal length density (HLD, g m^−1^ soil).

Soils were sampled from the well-mixed soils of each mesocosm in March, June, September, and December 2020. Furthermore, each soil sample was sieved through a 2 mm mesh to remove visible plant material and then air-dried prior to measurements of soil properties. Mycorrhizal-secreted proteins (GRSP) were determined by the Bradford method, and root biomass (RB) was determined by drying and weighing the sample [23,24]. Soil bulk density (BD) was measured by the core method. The pH was determined using a glass electrode meter in a 1:2.5 soil–water solution (*w*/*v*). Microbial biomass carbon (MBC) was measured by chloroform fumigation extraction, and soil organic matter (SOM) was measured by the dichromate oxidation with an external heating procedure. Total nitrogen (TN) and total phosphorus (TP) were measured by continuous flow injection analysis (FOSS Company, Hillerød, Denmark-FLAstar 5000 Analyzer). Soil nitrate nitrogen (NO_3_^−^-N) and ammonium nitrogen (NH_4_^+^-N) were determined by an automatic flow analyzer (SFA—Segmented Continuous Flow Analysis) [25]. Available potassium (AK) was measured by the ammonium acetate extraction flame method, and available phosphorus (AP) was measured by the Mo-Sb colorimetric method. Soil respiration activity was measured by the indigo phenol colorimetric method, soil phosphatase (S-ALP) activity by the disodium nitrobenzene phosphate method, and soil sucrase (S-SC) activity by the Cu^2+^ alkaline reduction assay method.

### 2.5. Data Analysis

Before the analysis of variance, the normality of distribution of all data was examined with Shapiro–Wilk’s test, while homogeneity of variance was tested with the Bartlett test. A one-way analysis of variance (ANOVA) was used to determine the significant differences between treatments, followed by the Tukey HSD test for multiple comparisons (*p* < 0.05). A two-way ANOVA was used to analyze the effect of different experimental treatments (E) and sampling periods (P), as well as the interactions (E × P), on soil respiration rates. The functions of exponential regression (Van’t Hoff Equation (1)) were used to fit the relationship between SR and soil temperature (ST). We also performed quadratic regression analyses of SR against SW, MBC, MBN, and RB using Equation (2) as follows:

*SR* = αe*^βST^*, Q_10_ = e^10*β*^ (1)

*SR* = a*A*^2^ + b*A* + c (2)

where SR is the soil respiration rate (μmol·m^−2^·s^−1^), ST is the soil temperature (°C), α is the soil respiration rate when the soil temperature is 0 °C, β is the temperature response coefficient in the exponential model between soil respiration and temperature, and Q_10_ is the temperature sensitivity (the increase in the soil respiration rate per 10 °C temperature) of soil respiration to temperature [26]. Here, a, b, and c are the regression coefficients, and A represents SW, MBC, MBN, and RB.

Considering that most of the variables were heteroscedastic, the Mantel test was used to measure the effects of root and soil variables (e.g., root biomass, mycorrhizal secretory proteins, pH, soil organic matter, and soil available nitrogen) on soil respiration under different AM fungi inoculations using the package ‘vegan’ in RStudio 4.2.1. The piecewise structural equation model (PSEM) in the RStudio package ‘piecewiseSEM’ was used to denote interaction pathways to investigate key factors and potential driving mechanisms affecting soil respiration. The Chi-Squared and Fisher’s C indices were smaller, indicating the best fitting and most explanatory ability of the model for variations in soil respiration. All statistical analyses were conducted with a significance level of 0.01 or 0.05 using RStudio 4.2.1 statistical software.

## 3. Results

### 3.1. Temporal Variation in Soil Respiration Under AM Fungi Inoculation

**Figure 1:**
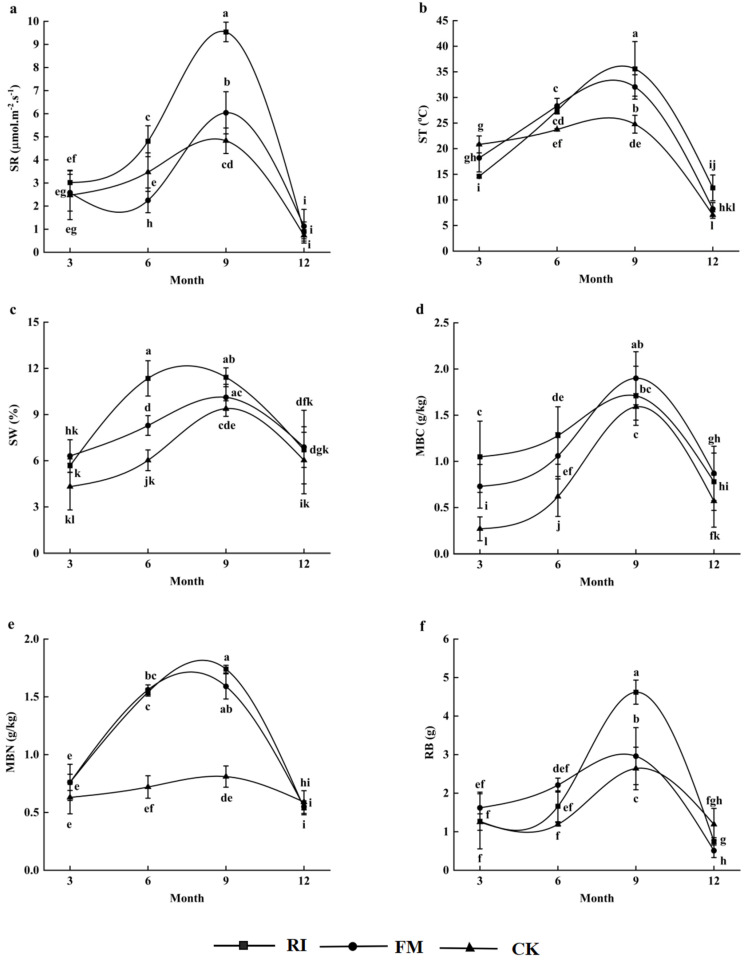
Seasonal dynamics of SR (**a**), ST (**b**), SW (**c**), MBC (**d**), MBN (**e**), and RB (**f**) under different experimental treatments. SR: soil respiration rate; ST: soil temperature; SW: soil water content; MBC: microbial biomass carbon; MBN: microbial biomass nitrogen; RB: root biomass; RI: *R. intraradices* addition; FM: *F. mosseae* addition; CK: no mycorrhizae addition. Columns are mean ± SE (standard error). Different lowercase letters indicate a significant difference in the experimental treatments (Tukey HSD, *p* < 0.05).

This study observed a significant effect of experimental treatments on the seasonal dynamics of soil respiration (F = 5.45, df = 2, *p* < 0.05; Table 1). The inoculation with RI (0.90–9.54 μmol·m^−2^·s^−1^) and FM (1.13–6.04 μmol·m^−2^·s^−1^) had a higher magnitude of seasonal variation in respiration rates compared with no mycorrhizae addition (0.73–4.83 μmol·m^−2^·s^−1^) in rocky desertification soils (Figure 1a). In particular, the average respiration rates (4.57 μmol·m^−2^·s^−1^) were higher in the RI inoculation than in the FM inoculation (3.00 μmol·m^−2^·s^−1^) (Figure 1a).

The soil respiration under the two AM fungi treatments and the control was significantly affected by sampling month (F = 27.88; df = 3; *p* < 0.01) (Table 1). They were all the highest in September (Figure 1a), which was closely associated with the highest soil temperature and water content in the month (Figure 1b,c). The lowest soil respiration rates in the RI and FM were in December when the soil temperature had minimum values (Figure 1a,b). In contrast, the minimum soil respiration rate was observed in December when soil water content was at its lowest value (Figure 1a,c).

The soil respiration also showed a similar seasonal variation with MBC, MBN, and RB under AM inoculation treatments (Figure 1d–f). For example, the soil respiration (3.53–7.79 μmol·m^−2^·s^−1^), MBC (1.17–1.81 g/kg), MBN (1.55–1.66 g/kg), and RB (1.64–3.78 g) were all significantly higher in wet seasons (June and September) than in dry seasons (March and December) under AM fungi inoculation treatments. Furthermore, in contrast with the control, the MBC, MBN, and RB had higher increases under RI (1.39–3.45 times) and FM (1.71–2.88 times) fungi inoculation in the wet seasons than in the dry seasons (0.89–2.30 times), which was consistent with the seasonal dynamics in the soil respiration. Thus, soil respiration rates can be significantly affected by AM fungi species, sampling month, and MBC, MBN, and RB variation (Figure 1 and Table 1).

### 3.2. Shift in Plant Growth, Microbial, and Soil Variables Under AM Fungi Inoculation

The plant growth, roots, microbial biomass, and soil physicochemical properties were significantly affected by AM fungi inoculation, but these effects varied among the two AM fungi treatments (Table 2; *p* < 0.05). The tree height (1.06–1.11 times), BDH (1.02–1.05 times), RB (1.52–1.73 times), RC (1.63–2.55 times), and HLD (1.17–2.11 times) significantly increased under two AM fungi inoculations compared with the control, while there was no difference in GRSP under three treatments (*p* > 0.05). The MBC (1.97–2.09 times) and MBN (1.63–1.68 times) were elevated by AM fungi treatments, while there was no difference in MBC and MBN between the two AM fungi treatments. The values of ST (1.14–1.18 times), SW (1.23–1.36 times), NO_3_^−^-N (1.09–1.84 times), NH_4_^+^-N (1.09–1.44 times), TN (2.32–2.57 times), TP (1.33–1.75 times), AP (1.39–1.43 times), AK (1.04–1.27 times), and SOC (1.25–1.30 times) were elevated by two AM fungi inoculation treatments, while BD (1.09–1.21 times) and pH (1.00–1.01 times) were reduced by AM fungi inoculation treatments. There was no significant difference in bulk density under RI and FM inoculation treatments. The RI inoculation treatment had the greatest increase in values (17.29–108.62%) for ST, SW, NO_3_^−^-N, NH_4_^+^-N, AP, SOC, MBC, and MBN in contrast with the control. In contrast, the highest increases (27.40–157.01%) were in TN, TP, and AK, and the lowest increases were in soil BD (10.83–32.82%) and pH (0.96.23–2.09%) under FM inoculation treatments. We also observed that the three treatments had different effects on soil enzyme activity. The S-ALP (1.40–2.39 times) and S-UE (1.36–1.82 times) increased under two AM fungi treatments. The S-ALP (133.88–145.46%) had the greatest increase under RI treatment compared with reference soils, and the S-ALP had a significant difference between the two AM fungi treatments. In contrast, the S-SC (17.65–27.27%) had the lowest increase under FM inoculation treatment. There was no significant difference between S-SC, S-ALP, and S-UE under the FM treatment compared with the control.

### 3.3. Linking Soil Respiration with Soil Variables, Microbial Biomass, and Root Biomass Under AM Fungi Inoculation

**Figure 2:**
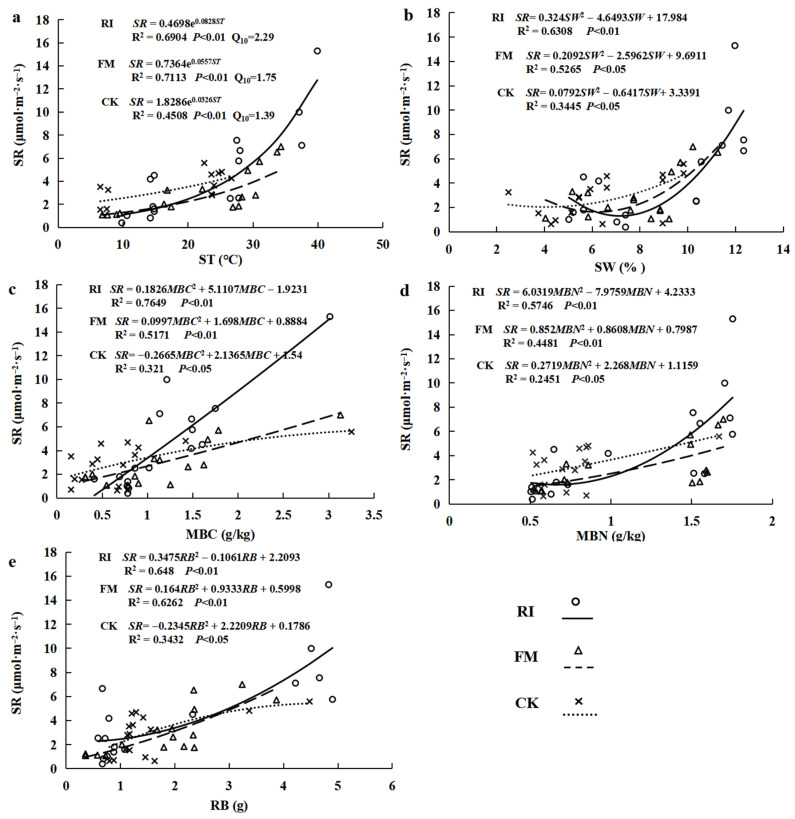
Regression analysis of ST (**a**), SW (**b**), MBC (**c**), MBN (**d**), and RB (**e**) affecting soil respiration under different experimental treatments. SR: soil respiration rate; ST: soil temperature; SW: soil water content; MBC: microbial biomass carbon; MBN: microbial biomass nitrogen; RB: root biomass; RI: *R. intraradices* addition; FM: *F. mosseae* addition; CK: no mycorrhizae addition.

Soil respiration increased exponentially with ST, SW, MBC, MBN, and RB under three treatments (Figure 2, *p* < 0.05). The ST has a higher explanation rate for soil respiration dynamics by FM (71.13%) inoculation than by RI (69.04%) inoculation treatment (Figure 2a). The sensitivity of soil respiration to ST was significantly higher under RI (Q_10_ = 2.29) and FM (Q_10_ = 1.75) fungi inoculation than under no mycorrhizae addition (Q_10_ = 1.39). The SW was higher in RI (8.79 ± 2.82%) and FM (7.90 ± 1.97%) fungi inoculation than in the control (6.44 ± 2.27%) (Figure 1c, *p* < 0.01), and the explanation rates for soil respiration variation were 63.08% and 52.65%, respectively (Figure 2b). The MBC, MBN, and RB were higher under AM fungi treatments than under no mycorrhizae addition (Table 2; *p* < 0.05). Compared with the control, the MBC, MBN, and RB had a higher level of explanation rates for soil respiration under RI treatments (76.49%, 57.46%, and 64.80%, respectively) than FM (51.71%, 44.81%, and 62.62%, respectively) inoculation treatment (Figure 2c–e). Therefore, the increased ST, SW, MBC, MBN, and RB under AM fungi treatments had significant effects on soil respiration.

**Figure 3:**
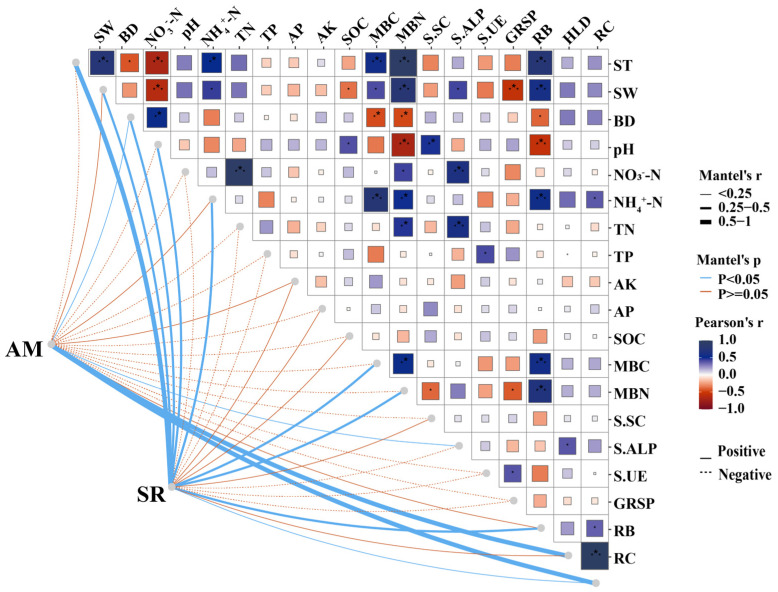
Mantel test analysis showing the effects of plant and soil variables on soil respiration under AM fungi inoculation treatments. Asterisks indicate significant correlations (*** *p* ≤ 0.001, ** *p* ≤ 0.01, and * *p* ≤ 0.05). ST: soil temperature; SW: soil water content; BD: soil bulk density; NO_3_^−^-N: nitrate nitrogen; NH_4_^+^-N: ammonium nitrogen; TN: total nitrogen; TP: total phosphorus; AP: available phosphorus; AK: available potassium; SOC: soil organic carbon; MBC: microbial biomass carbon; MBN: microbial biomass nitrogen; S-SC: soil sucrase; S-ALP: soil phosphatase; S-UE: soil urease enzyme; GRSP: mycorrhizal-secreted proteins; RB: root biomass; RC: root colonization; HLD: hyphal length density; AM: *R. intraradices* and *F. mosseae* additions; SR: soil respiration rate.

The AM fungi-mediated soil properties, microbial biomass, and root biomass had significant impacts on soil respiration rates (Figure 3). The results of the Mantel test showed that the values of RB, RC, HLD, MBC, MBN, ST, SW, BD, pH, and NH_4_^+^-N were extremely positively correlated with soil respiration under AM fungi inoculation (*p* < 0.01 or 0.05), whereas the concentrations of NO_3_^−^-N, TN, TP, S-ALP, S-UE, and GRSP showed a negative relationship with soil respiration rates. No significant correlations of soil respiration rates were found with AK, AP, SOC, and S-SC. We also found that the BD, S-ALP, RC, and HLD were positively correlated with AM fungi species (*p* < 0.01 or 0.05). In contrast, there was no significant correlation of soil respiration with SW, NH_4_^+^-N, AK, and RB, but there was a negative correlation with ST, pH, NO_3_^−^-N, TN, TP, AP, SOC, MBC, MBN, S-SC, S-UE, and GRSP.

### 3.4. Pathways of Plant, Microbial, and Soil Properties Regulating Soil Respiration Under AM Fungi Inoculation

**Figure 4:**
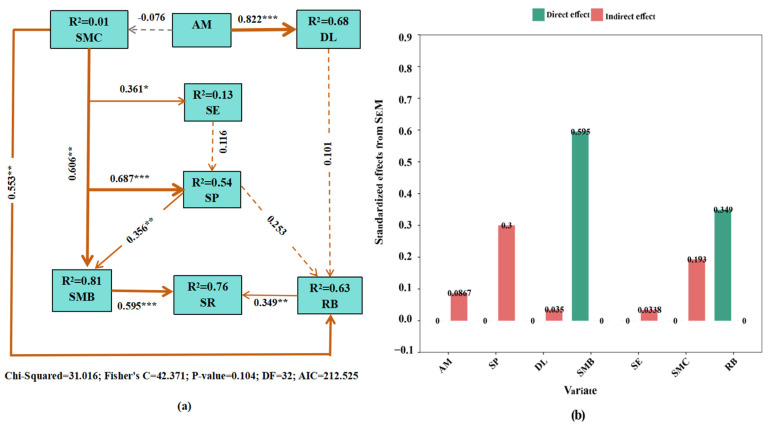
The structural equation model (SEM) showing direct and indirect pathways of plant, microbial, and soil variables affecting soil respiration. (**a**) SEM shows the direct and indirect effects of microbial biomass and the root system on soil respiration. (**b**) The standardized effects (direct and indirect) are deduced from the structural equation model. Continuous arrows represent significant relationships, while dashed arrows represent non-significant relationships. The numbers next to the arrows denote the path coefficients. Asterisks represent significant correlations (*** *p* ≤ 0.001, ** *p* ≤ 0.01, and * *p* ≤ 0.05). Orange arrows represent positive relationships, while gray arrows represent negative relationships. The thickness of the arrows represents the size of the standardized path coefficients. SMC: soil microclimate; DL: colonization level; SE: soil enzyme activity; SP: soil physicochemical properties; SMB: soil microbial biomass; RB: root biomass.

The changes in SMC, DL, SE, SP, SMB, and RB directly or indirectly impacted soil respiration rates under AM fungi-mediated conditions (Figure 4). The SEM captured 76% of the variation in soil respiration (Figure 4a). The changes in SMB (*R*^2^ = 0.81; path coefficient = 0.60; *p* < 0.001) and RB (*R*^2^ = 0.63; path coefficient = 0.35; *p* < 0.01) under AM fungi inoculation directly and positively affected soil respiration. The SP had the strongest positive indirect effect on soil respiration through affecting SMB (path coefficient = 0.36, *p* < 0.001), explaining 30% of the variation in soil respiration. The SMC positively and indirectly affected soil respiration by influencing SE (path coefficient = 0.36, *p* < 0.05), SP (path coefficient = 0.69, *p* < 0.001), SMB (path coefficient = 0.61, *p* < 0.01), and RB (path coefficient = 0.55, *p* < 0.01). In contrast, the SE indirectly affected soil respiration by affecting SMB and RB. Furthermore, the SMB (59.50%) and RB (34.90%) had the greatest direct effect on soil respiration; the indirect effects of SP (30.00%), SMC (19.30%), DL (3.50%), and SE (3.38%) ranked second (Figure 4b).

## 4. Discussion

### 4.1. Impact of AM Fungi Inoculation on Soil Respiration

Our results confirmed that the soil respiration increased under two AM fungi inoculations compared with no AM fungi addition. This may be attributed to the fact that AM fungi can form a complicated soil mycelium network system that accelerates the absorption of soil water and nutrients by plant roots [27,28], thereby promoting root growth and root respiration. Furthermore, we found that two fungi treatments had a higher increase in soil total phosphorus and available phosphorus compared with the control [29]. This may be due to the mycelium secretions of AM fungi that can promote phosphorus mineralization and dissolution and stimulate soil phosphatase activity, thus affecting soil respiration [30]. Therefore, AM fungi may stimulate soil respiration through promoting plant growth, nutrient availability, and soil enzyme activity.

The effects of AM fungi inoculation on soil respiration varied with the species. We observed that RI fungi inoculation had higher root colonization and hyphal length density compared with FM fungi inoculation, which may have a greater increase in soil respiration. This may be attributed to the fact that RI fungi had greater symbiotic ability with plants to form a mycelium bridge that improves the root water and nutrient absorption as well as accelerates the transfer and accumulation of carbon compounds [31,32]. We also observed a higher potential of RI inoculation in increasing the activities of sucrose enzyme, phosphatase, and urease compared with the FM inoculation. This finding is in agreement with An et al. [33], who found that RI fungi can penetrate into the soil micropores via a mycelium network to release organic acids and enzyme substances and to dissolve insoluble potassium and phosphorus into the soil, which would directly and indirectly promote soil respiration [34]. The RI fungi inoculation can also form typical arbuscular structures to penetrate into the interior of plant root cells, assimilate many more nutrients into the mycelium, and improve the utilization efficiency of carbon and nitrogen nutrients of plants from soils [35]. Our data suggested that two AM fungi species had different effects on soil respiration, which may be closely associated with their disparate arbuscular structure and the ability to regulate root and microbial growth as well as soil nutrient availability.

The increase in soil water content under AM fungi inoculation significantly enhanced the explanation of soil respiration, which may be due to their role in the mobilization of nutrients for microbes. During the rainy season, a higher mycelium growth of AM fungi loosens and ventilates the soil, increasing water penetration into the soil, and the increase in soil moisture accelerates the nutrient uptake by plants [36,37]. In particular, these changes may stimulate root and microbial activities, thereby increasing soil respiration. On the other hand, the higher soil temperature and humidity in the wet season can favor litter decomposition and organic matter degradation to provide carbon and nitrogen nutrients for root and microbial growth and thus accelerate soil respiration [38,39]. In the wet season, RI fungi treatment had a higher soil respiration rate than FM fungi treatment, which may be due to a greater improvement in water permeability, antioxidant capacity, the nutrient metabolism efficiency of plants, and the endogenous hormone balance ability of plants, which all stimulate the activity of plant roots and rhizosphere microorganisms [40]. Therefore, the effects of AM fungi inoculation on seasonal change in soil respiration, primarily through mediating mycelium growth, affect plant root, microbial, soil microclimate, and nutrient conditions in the rocky desertification habitat.

### 4.2. Impact of Plant and Soil Variables on Soil Respiration

AM fungi play a crucial role in regulating soil respiration through mediating the above-ground (i.e., tree height and diameter at breast height) and below-ground (i.e., root colonization and hyphal length density) growths. In this study, the AM fungi inoculation significantly increased the plant height, which stimulated the root respiration. This may be due to AM fungi forming symbiotic relationships with plants, promoting plant growth and the input of photosynthetic products into the soil [41], thus stimulating root and microbial respiration. Our study also observed that AM fungi inoculation significantly increased root colonization and hyphal length density, as well as root colonization, which was positively correlated with soil respiration in agreement with Khan et al. [42]. In a nutrient-deficient environment, AM fungi can accelerate the root colonization and hyphae length density, which expands the range of mycorrhizal activity and improves the effectiveness and utilization of nitrogen, phosphorus, and potassium nutrients [43], thus increasing the respiration of plant roots and microorganisms. Therefore, AM fungi can significantly affect soil respiration by promoting plant growth and root colonization.

In this study, we found that the soil phosphatase enzyme activity increased under AM fungi addition compared with the control soil and positively correlated with soil respiration, which is in agreement with Wang et al. [44]. AM fungi can secrete carbohydrates and other compounds from plants into the mycorrhizal network, providing a rich carbon source for phosphate-solubilizing bacteria. This would promote the production of phosphatase and organic acid to enhance the decomposition of organic phosphorus compounds [45], thus increasing the soil respiration rate. We also found the same increased changes in urease under AM fungi treatments. It is possible that the metabolic activities of AM fungi can enrich some beneficial microorganisms that directly increase the content of soil urease and the increased diversity of rhizosphere microbial communities [46], which affects soil respiration. Therefore, AM fungi may affect soil respiration by promoting soil enzyme activity.

AM fungi-mediated change in soil variables has a crucial effect on soil respiration [47]. Our study found that inoculation of AM fungi reduced soil bulk density. The mycelium of AM fungi can penetrate and loosen the soil to increase the porosity, thus improving the aeration and water permeability of the soil. This may promote plant root growth, which has positive direct impacts on soil respiration [48,49]. We also observed that AM fungi inoculation increased the levels of soil carbon components and air permeability, which facilitated soil respiration. This may be due to a high secretion of energy-rich carbon compounds by the AM mycelium, which ultimately enhances soil microbial respiration [50]. Furthermore, AM fungi-mediated changes increased the concentration of soil nitrogen pools, which stimulated soil respiration. The extracellular hyphae of AM fungi increased the level of nitrate nitrogen, which favors the growth of plant roots and soil microbes [51]. Therefore, AM fungi-mediated changes in soil variables can exert an important effect on soil respiration.

### 4.3. Impact of Microbial and Root Biomass on Soil Respiration

This study observed a higher increase in soil respiration due to increased root and microbial biomass that were accelerated by AM fungi colonization. Compared with the control, AM fungi inoculation increased root biomass and the interpretation intensity of soil respiration, and root biomass was positively correlated with soil respiration. This is probably due to AM fungi forming arbuscular structures by invading plant root peridermal cells, thus enhancing the plant’s ability to absorb soil nutrients, increasing root growth and root biomass [35]. This can promote root respiration and microbial respiration. Furthermore, AM fungi symbiosis with plants can form mycelial bridges to promote the growth of the roots and the absorption of mineral elements [17], which increases the root respiration. We also found that the increase in microbial biomass carbon and nitrogen increased the interpretation intensity of soil respiration under AM fungi inoculation. This may be due to the AM fungi hyphae secretions being coupled with soil microbial residues to form an excitation effect, which increases soil carbon and nitrogen content, thereby increasing the soil microbial respiration [52]. Therefore, AM fungi may directly promote soil respiration by increasing microbial and root biomass.

## 5. Conclusions

This study found that two AM fungi species had a significant increase in soil respiration in the rocky desertification area. This may be attributed to their positive effects on plant growth, microclimates, nutrient pools, soil enzyme activities, and soil microbial biomass. Furthermore, a higher soil respiration was found under RI fungi inoculation compared with FM fungi inoculation, which may be due to the different modifications of AM species on soil microbial and physicochemical variables. In particular, the increased soil respiration was mainly driven by microbial biomass carbon, microbial biomass nitrogen, and root biomass. Therefore, the results indicate that two AM fungi species have different positive effects on soil respiration, primarily via the differentiated modification of microbial and root biomass in the rocky desertification area. These results further the understanding of the impact of the interactions between plants, AM fungi, and soil properties on the soil carbon cycle in rocky desertification areas.

## Figures and Tables

**Table 1:** F-values of ANOVA showing effects of experimental treatments (E) and sample months (S) on SR, ST, SW, MBC, MBN, and RB.

Source	df	SR	ST	SW	MBC	MBN	RB
E	2	5.45 **	10.42 **	14.30 **	2.65 **	130.83 **	1.55
S	3	27.88 **	226.64 **	35.60 **	8.11 **	244.96 **	22.29 **
E × S	6	2.13	11.31 **	3.22	0.33	37.12 **	2.58

E: experimental treatments; S: sampling month; SR: soil respiration rate; ST: soil temperature; SW: soil water content; MBC: microbial biomass carbon; MBN: microbial biomass nitrogen; RB: root biomass. Significant levels: ** *p* < 0.01.

**Table 2:** Plant growth, microbial, and soil variables, as well as mycorrhizal colonization under three treatments in the desertification soils.

Treatment	RI	FM	CK
ST (°C)	22.49 ± 10.16 a	21.70 ± 9.77 a	19.09 ± 7.42 a
SW (%)	8.79 ± 2.82 a	7.90 ± 1.97 ab	6.44 ± 2.27 b
BD	1.58 ± 0.18 a	1.44 ± 0.13 ab	1.74 ± 0.88 b
pH	7.68 ± 0.31 a	7.57 ± 0.27 a	7.68 ± 0.31 a
NO_3_^−^-N (mg/g)	4.26 ± 2.93 a	2.53 ± 1.86 ab	2.32 ± 1.65 b
NH_4_^+^-N (g/kg)	4.42 ± 2.24 a	3.35 ± 2.14 ab	3.07 ± 0.93 b
TN (mg/kg)	2.48 ± 1.74 a	2.75 ± 1.99 a	1.07 ± 0.41 b
TP (mg/g)	1.26 ± 0.38 b	1.66 ± 0.42 a	0.95 ± 0.12 c
AP (mg/g)	5.61 ± 1.14 a	5.42 ± 1.73 a	3.91 ± 1.62 b
AK (mg/g)	3.05 ± 1.78 a	3.72 ± 1.73 a	2.92 ± 1.23 a
SOC (g/kg)	6.52 ± 1.93 a	6.29 ± 1.24 ab	5.02 ± 1.28 b
MBC (g/kg)	1.21 ± 0.62 a	1.14 ± 0.70 a	0.58 ± 0.74 b
MBN (g/kg)	1.14 ± 0.53 a	1.11 ± 0.49 a	0.68 ± 0.13 b
S-SC (nmol/g/h)	2.66 ± 0.59 a	2.28 ± 0.52 b	2.32 ± 0.06 b
S-ALP (nmol/g/h)	2.63 ± 0.20 a	1.54 ± 0.41 b	1.10 ± 0.11 b
S-UE (nmol/g/h)	3.58 ± 2.37 a	2.67 ± 1.39 ab	1.97 ± 0.81 b
GRSP (mg/g)	4.36 ± 0.60 a	4.38 ± 0.61 a	4.35 ± 0.63 a
RB (g)	2.07 ± 1.83 a	1.82 ± 1.00 a	1.20 ± 0.97 b
RC (%)	90.43 ± 5.07 a	57.54 ± 2.77 b	35.40 ± 1.71 c
HLD (m/g)	4.01 ± 0.45 a	2.23 ± 0.25 b	1.90 ± 0.08 c
H (cm)	77.67 ± 15.44 a	74.19 ± 15.96 b	70.04 ± 17.07 c
BDH (mm)	7.50 ± 1.89 a	7.23 ± 2.00 b	7.11 ± 2.09 c

ST: soil temperature; SW: soil water content; BD: soil bulk density; NO_3_^−^-N: nitrate nitrogen; NH_4_^+^-N: ammonium nitrogen; TN: total nitrogen; TP: total phosphorus; AP: available phosphorus; AK: available potassium; SOC: soil organic carbon; MBC: microbial biomass carbon; MBN: microbial biomass nitrogen; S-SC: soil sucrase; S-ALP: soil phosphatase; S-UE: soil urease enzyme; GRSP: mycorrhizal-secreted proteins; RB: root biomass; RC: root colonization; HLD: hyphal length density; H: tree height; BDH: diameter at breast height; RI: *R. intraradices* addition; FM: *F. mosseae* addition; CK: no mycorrhizae addition. Different lowercase letters indicate a significance in the different experimental treatments (Tukey HSD, *p* < 0.05).

## Data Availability

The data that support the findings of this study are available from the corresponding author upon reasonable request.
